# SNP 1772 C > T of HIF-1α gene associates with breast cancer risk in a Taiwanese population

**DOI:** 10.1186/s12935-014-0087-7

**Published:** 2014-09-26

**Authors:** Chih-Jen Huang, Shi-Long Lian, Ming-Feng Hou, Chee-Yin Chai, Yi-Hsing Yang, Sheng-Fung Lin, Hsueh-Wei Chang

**Affiliations:** Graduate Institute of Medicine, College of Medicine, Kaohsiung Medical University Faculty of medicine, Kaohsiung Medical University, Kaohsiung, Taiwan; Department of Radiation Oncology, Kaohsiung Medical University Hospital, Kaohsiung, Taiwan; Institute of Clinical Medicine, Kaohsiung Medical University, Kaohsiung, Taiwan; Kaohsiung Municipal Ta-Tung Hospital, Kaohsiung, Taiwan; Cancer Center, Kaohsiung Medical University Hospital, Kaohsiung Medical University, Kaohsiung, Taiwan; Department of Pathology, Kaohsiung Medical University Hospital, Kaohsiung Medical University, Kaohsiung, Taiwan; Institute of Biomedical Sciences, National Sun Yat-Sen University, Kaohsiung, Taiwan; Department of Pathology, Kaohsiung Medical University Hospital, Kaohsiung Medical University, Kaohsiung, Taiwan; School of Pharmacy, Kaohsiung Medical University, Kaohsiung, Taiwan; Department of Medical Oncology, Kaohsiung Medical University Hospital, Kaohsiung Medical University, Kaohsiung, Taiwan; Translational Research Center, Kaohsiung Medical University Hospital, Kaohsiung Medical University, Kaohsiung, Taiwan; Institute of Medical Science and Technology, National Sun Yat-sen University, Kaohsiung, Taiwan; Department of Biomedical Science and Environmental Biology, Kaohsiung Medical University, Kaohsiung, Taiwan

**Keywords:** HIF-1α, SNPs, Breast cancer, Association study, Survival

## Abstract

**Background:**

Hypoxia inducible factor 1α (HIF-1α) is a stress-responsive transcription factor to hypoxia and its expression is correlated to tumor progression and angiogenesis. Several single nucleotide polymorphisms (SNPs) of HIF-1α gene in the oxygen-dependent degradation (ODD) domain was reportedly associated with increased HIF-1α activity.

**Results:**

In this study, we focused on the relationship between SNP 1772 C > T (rs11549465) of HIF-1α gene and its breast cancer risk, as well as its correlation with HIF-1α expression and tumor angiogenesis. Ninety six breast cancer patients and 120 age-matched controls were enrolled. We found that 1772 T allele of HIF-1α gene was associated with increased breast cancer risk (adjusted OR = 14.51; 95% CI: 6.74-31.24). This SNP was not associated with clinicopathologic features of angiogenesis such as VEGF activity and the micro-vessel density and survival of breast cancer patients.

**Conclusion:**

Taken together, the 1772 C > T of HIF-1α gene is a potential biomarker for breast cancer susceptibility.

## Background

Single nucleotide polymorphisms (SNPs), the most common variants in human genome [1], are popular biomarkers for disease/cancer prediction and therapeutic evaluation [2-8]. Most SNPs have been reported to be associated with breast cancer [9-11], however, other SNPs are still potential to be associated with breast cancer.

Tumor hypoxia is common in tumorigenesis. Hypoxia inducible factor-1 (HIF-1) is a crucial transcription factor in cellular response to tumor hypoxia and is considered as an adverse prognostic factor in breast cancers [12-14]. Additionally, the HIF-1α isoform is the oxygen-regulated component that controls HIF-1 activity [15]. The degradation of HIF-1α depends on prolyl hydroxylation. Under normoxic status, oxygen-dependent prolyl hydroxylases [16,17] may hydroxylate the HIF-1α on proline residues 402 and 564 located in the oxygen-sensitive degradation domain (ODD, encoded by codons 401–603) of HIF-1α. In contrast, degradation of HIF-1α is suppressed under hypoxic status. Therefore, the SNPs located at several proline residues of HIF-1α gene in breast cancer association are potential to modulate the HIF-1α activity.

Recent studies demonstrated that another SNP located in ODD of HIF-1α, 1772 C > T (rs11549465), may lead to an amino acid change from proline 582 to serine (P582S) and are reportedly associated with renal [18,19], head and neck [20], prostate [21], lung [22], and pancreatic [23] cancers. Meta-analysis from 34 case–control studies also reported that SNP 1772 C > T (P582S) of HIF-1α gene is significantly associated with breast cancer risk in many countries [24]. However, the association of SNP 1772 C > T (rs11549465) of the HIF-1α gene to breast cancer remains unclear in a Taiwanese population.

The purpose of this study is to investigate the association between SNP 1772 C > T of the HIF-1α gene in breast cancer patients and healthy control subjects. Furthermore, HIF-1 has been reported to transactivate many oxygen responsive genes such as vascular endothelial growth factor (VEGF) [25]. Therefore, the relationships between genotypes of SNP 1772 C > T of HIF-1α gene and the clinicopathologic characteristics, the immunostaining expression levels of HIF-1α and VEGF, and clinical outcomes of breast cancer are also addressed in this study.

## Methods and materials

### Patient characteristics and control subjects

Between 1991 and 2001, a total of 96 randomly-selected female patients with breast cancer at Kaohsiung Medical University Hospital, Kaohsiung, Taiwan, were enrolled in this study. All patients underwent a standard modified radical mastectomy. Ninety-four patients (94/96, 98%) received adjuvant systemic chemotherapy with 6 cycles of 5-fluorourcil, doxorubicin and cyclophosphamide. After completion of chemotherapy, all patients received hormone therapy with tamoxifen and 92 patients (92/96, 96%) received radiation therapy. The principle of treatment was followed as described previously [26]. We collected clinical data including clinical stage, treatment outcomes and follow-up status. Controls were recruited from 120 healthy female without a history of cancer and matched to the breast cancer patients by sex and age.

### DNA extraction and PCR-RFLP

Genomic DNA was isolated from paraffin-embedded tumor tissues of surgical specimens and peripheral blood of 120 normal controls as described [27,28]. The sequence of primers for HIF-1α is as follows: forward 5′-AGGACACAGATTTAGACTTGG-3′ and reverse 5′-GGAATACTGTAACTGTGCTTTG-3′. PCR reaction mixture (10 μl) contained 1 μl of 10× PCR buffer, 0.3 μl of 50 mM MgCl_2_, 0.2 μl of 10 mM dNTP each, 0.6 μl DMSO, 0.14 μl of Taq enzyme, 0.12 μl of 350 μg/ml primers mix (1:1), 2 μl DNA extracts and 5.64 μl distilled water. PCR was performed with the following protocol: 94°C (1 min); 4 cycles of 94°C (15 s), 64°C (15 s), 70°C (8 s); 4 cycles of 94°C (15 s), 61°C (15 s), 70°C (8 s); 4 cycles of 94°C (15 s), 58°C (15 s), 70°C (8 s); 60 cycles of 94°C for (15 s), 55°C (15 s), 70°C (8 s); 94°C (1 min) and 60°C (5 min). The available restriction enzyme for HIF-1α 1772 C > T (rs11549465) was retrieved from the SNP-RFLP freeware [29-31]. PCR products were digested with the *Hph I* restriction enzyme (NEB) at 37°C for overnight and then they were subjected to 3% agarose electrophoresis and stained with SYBR Safe™ DNA gel stain (Invitrogen) for visualization of the PCR-restriction fragment length polymorphism (PCR-RFLP) patterns.

### Sequencing

Typical patterns of genotyping by PCR-RFLP have confirmed by sequencing. DNA amplicon from PCR reaction was purified using a MiniElute PCR purification kit (Qiagen) [28] for commercial sequencing.

### Immunohistochemical analyses of HIF-1α and VEGF proteins

Streptoavidin-biotin based immunohistochemical staining (IHC) was performed to detect HIF-1α and VEGF protein levels as previously described [32]. Immunoreactivity of HIF-1α was located in both nuclei and cytoplasm. Using a semiquantitative scale described previously [33], the HIF-1α expression were classified as follows: 1+, nuclear staining in less than 1% of cells; 2+, nuclear staining in 1-10% of cells and/or with weak cytoplasmic staining; 3+, nuclear staining in 10-50% of cells and/or with distinct cytoplasmic staining; 4+, nuclear staining in more than 50% of cells and/or with strong cytoplasmic staining. For further analysis, we defined two groups of low and high HIF-1α expression: 1+ or 2+ staining pattern regarded as low expression, and 3+ or 4+ staining pattern as high expression. VEGF expression was assessed according to the intensity of cytoplasmic staining as described previously [32]. VEGF expression was detected tumor cells in a distinct and strongly cytoplasmic staining. VEGF staining was defined as four grades as follows: no staining, weak, distinct and strong cytoplasmic staining. Distinct and strong cytoplasmic staining was defined as high VEGF and negative or weak cytoplasmic staining was defined as low VEGF expression.

### Immunohistochemical analysis for microvessel detection

Microvessel density (MVD) represents tumor angiogenesis by using immunostaining of endothelial cells with monocloncal antibody, recognizing the CD31 endothelial glycoprotein. Each slide was scanned at low magnification (× 100) to identify the four areas of high density of microvessels (hotspots). The number of stained vessels per in each hotspot was counted at high power fields (× 400). Any stained endothelia cell was considered as a countable single microvessel. Large vessels with thick muscular walls were excluded. MVD was classified as either low (≦35.0) or high (>35.0/high power field (HPF)); 35.0 was the median value.

### Statistical analysis

Statistical significance was evaluated by the chi-square test and Fisher exact test. Overall survival curves were analyzed by the Kaplan-Meier method, and differences between the curves were analyzed by log-rank test. The *p* values smaller than 0.05 are regarded as significance.

## Results

In Table 1, the mean age of the breast cancer patients was 46.5 years (range 19–73 years), and this was 44.6 years for controls (range 21–77 years). There was no significant difference between breast cancer patients and controls in age (*p* = 0. 22).

**Table 1:** **HIF-1**α **1772 C > T genotype and allele frequencies in breast cancer patients and control subjects**

**Parameters**	**Breast cancer patients**	**Control subjects**	***p*** **value** ^**a**^	**Crude** ***OR***	**Adjusted** ***OR*** ^**b**^	***p*** **value**	**95% CI**
Age	46.5 ± 9.9 (19–73)	44.6 ± 11.5 (21–77)	0.224				
CC (%)	53 (55%)	116 (97%)		1.00	1.00		
CT (%)	21 (22%)	0 (0%)					
TT (%)	22 (23%)	4 (3%)	<0.001	12.04	11.33	<0.001	3.70-34.72
CT/CC (%)	74 (77%)	116 (97%)		1.00	1.00		
TT (%)	22 (23%)	4 (3%)	<0.001	8.62	8.31	<0.001	2.74-25.25
CC (%)	53 (55%)	116 (97%)		1.00	1.00		
CT/TT (%)	43 (45%)	4 (3%)	<0.001	23.53	23.23	<0.001	7.92-68.09
C genotype (%)	127 (66%)	232 (97%)		1.00	1.00		
T genotype (%)	65 (34%)	8 (3%)	<0.001	14.84	14.51	<0.001	6.74-31.24

^a^Comparisons were performed by Chi-Square test.

^b^Adjusted by age by conditional logistic regression analysis.

*OR* = odds ratio; CI = Confidence interval.

**Figure 1:**
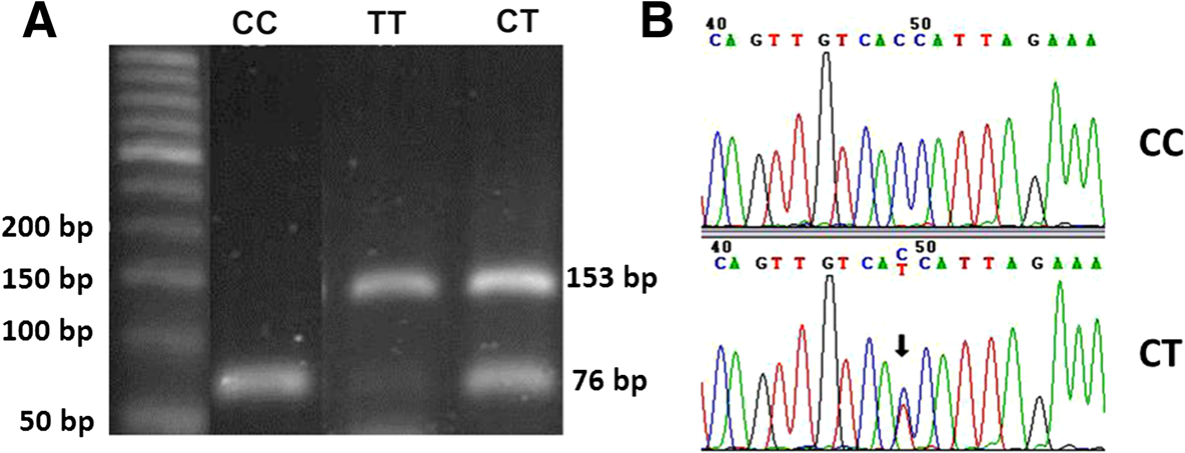
**PCR-RFLP genotyping and sequencing of SNP 1772 C > T of HIF-1α** **gene. (A)** PCR-RFLP genotyping of SNP 1772 C > T of HIF-1α gene (76 bp, C-allele; 153 bp, T-allele) in formalin-fixed, paraffin-embedded breast cancer tissues. **(B)** Sequence chromatograms of PCR-RFLP product contained SNP 1772 C > T of HIF-1α gene. Arrow indicated location of 1772 C > T.

In Figure 1A, RFLP results demonstrated that CC genotype yielded one band (76 base pairs), CT genotype yielded two bands (76 bp, C-allele; 153 bp, T allele) and TT genotype yielded one band (153 bp). The corresponding genotypes of homozygous and heterozygous patterns from PCR-RFLP had confirmed by DNA sequence analysis (Figure 1B).

Based on PCR-RFLP analysis, the genotype distribution of control group was 116 CC (97%), 0 CT (0%) and 4 TT (3%). In contrast, the genotype distributions of breast cancer patients were 53 CC (55%), 21 CT (22%), and 22 TT (23%). The genotype distribution in breast cancer patients differed significantly from that of controls (*p* < 0.001). The allele frequencies in controls and cancer patients were 232 C (97%)/8 T (3%) and 127 C (66%)/65 T (34%), respectively. The T-allele distribution in breast cancer patients differed significantly from that of controls (*p <* 0.001, adjusted *OR* = 14.51).

**Figure 2:**
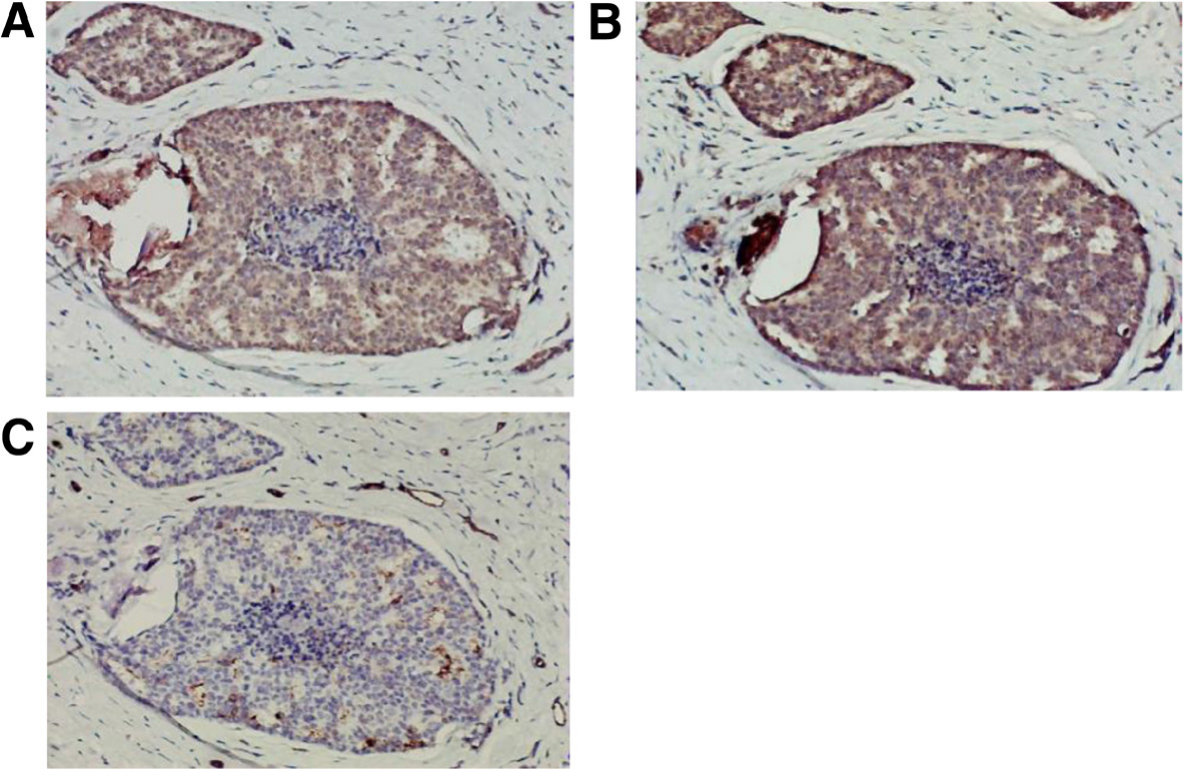
**Expression of (A) HIF-1α** **, (B) VEGF and (C) CD34 for microvessel density (200×) of a 34 year-old female patient with T2N1M0 breast cancer.**

Immunoreactivity of HIF-1α was distributed in both nuclei and cytoplasm (Figure 2A). VEGF expression was measured by its cytoplasmic staining (Figure 2B). Microvessel density (MVD) representing tumor angiogenesis was measured by immunostaining of CD31 endothelial glycoprotein (Figure 2C).

When connecting the results of these stainings with HIF-1α genotypes with clinicopathological analysis (Table 2), there were no significant correlation between 1772 C > T genotypes (CC, CT and TT) of HIF-1α gene and age (*p* = 0.117), T-stage (*p* = 0.303), N-stage (*p* = 0.936), local recurrence (*p* = 0.817), distant metastasis (*p* = 0.572), HIF-1α expression (*p* = 0.311), VEGF expression (*p* = 0.375) and microvessel density (*p* = 0.211).

**Table 2:** **Clinicopathologic characteristics, clinical outcomes in breast cancer patients with different HIF-1**α **1772 C > T genotypes**

**Genotype**	**CC (%)**	**CT (%)**	**TT (%)**	***p*** **value**
Case number	53 (55%)	21 (22%)	22 (23%)	
Age				0.117^a^
Mean ± SD (years)	46.5 ± 9.7	43.1 ± 10.3	49.4 ± 9.6	
Range (years)	27 ~ 68	19 ~ 62	31 ~ 73	
Laterality				0.463^b^
Left	26 (52%)	10 (20%)	14 (28%)	
Right	27 (58%)	11 (24%)	8 (17%)	
T-stage				0.303^b^
T1 or T2	35 (53%)	13 (20%)	18 (27%)	
T3 or T4	18 (60%)	8 (27%)	4 (13%)	
N-stage				0.936^b^
Node negative	14 (58%)	5 (21%)	5 (21%)	
Node positive	39 (54%)	16 (22%)	17 (24%)	
HIF-1α expression				0.311^b^
Low	34 (51%)	15 (22%)	18 (27%)	
High	19 (66%)	6 (21%)	4 (14%)	
VEGF expression				0.375^b^
Low	18 (62%)	7 (24%)	4 (14%)	
High	35 (52%)	14 (21%)	18 (27%)	
Microvessel density				0.211^b^
Low	32 (63%)	8 (16%)	11 (22%)	
High	21 (47%)	13 (29%)	11 (24%)	

^a^by ANOVA test.

^b^by Chi-Square test.

**Figure 3:**
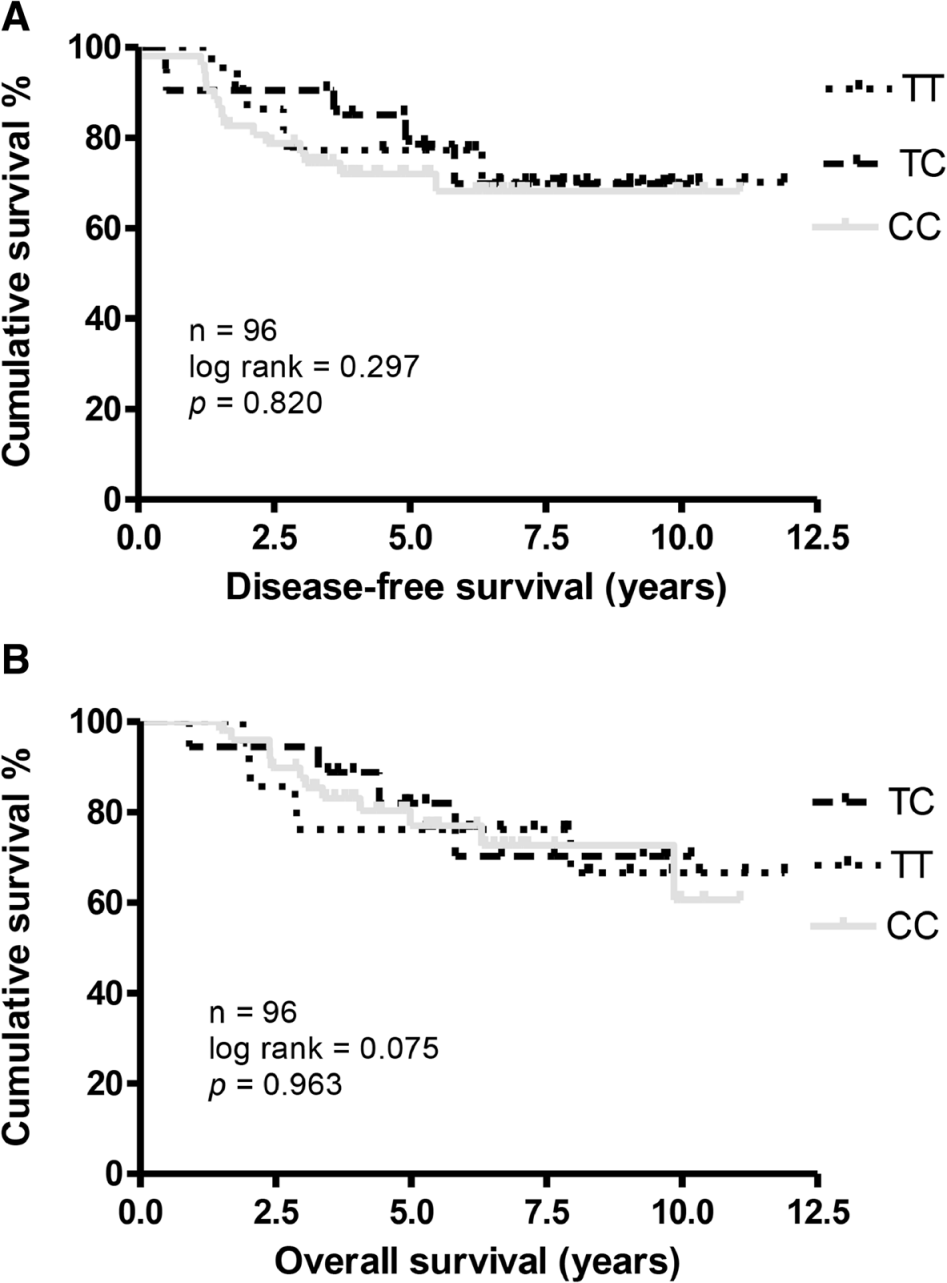
**Kaplan-Meier disease-free and overall survival curves in breast cancer patients with different genotypes (1772 C > T) of HIF-1α** **gene. (A)** Disease-free survival. **(B)** overall survival curves.

In Table 3, the multi-variable analyses in the determination of risk factors of disease-free survival and overall survival indicated that T-stage (Exp. (B) = 4.7270, *p* < 0.001) and microvessel density (Exp. (B) = 2.6082, *p* < 0.05) were the most influential factors (Table 3). However, the SNP 1772 C > T genotypes of HIF-1α gene were not correlated with the disease-free survival (*p* = 0.35, Cox regression) and overall survival (*p* = 0.59, Cox regression) by multi-variable analyses. Similarly, Kaplan-Meier analysis (Figures 3A and 3B) also showed a nonsignificant impact of 1772 C > T genotypes of HIF-1α gene on disease-free survival (*p* = 0.820, Log-Rank test) and overall survival curves (*p* = 0.963, Log-Rank test), respectively.

**Table 3:** **Multivariate analysis of the risk factors on disease-free and overall survival in the 96 breast cancer patients**

**Variable** ^**a**^	**SE**	***p*** **value** ^**b**^	**Exp. (B)**	**95% CI of Exp. (B)**	
Disease-free survival time					
Age	0.0253	0.1280	0.9622	0.9156	~1.0112
1772 C > T genotype	0.2715	0.3527	1.2871	0.7559	~2.1913
T-stage	0.4033	**0.0001**	4.7270	2.1445	~10.4196
N-stage	0.5291	0.9816	1.0122	0.3588	~2.8554
Microvessel density	0.4877	**0.0493**	2.6082	1.0028	~6.7837
VEGF expression	0.5858	0.2901	1.8584	0.5895	~5.8587
HIF-1α expression	0.4346	0.0732	2.1784	0.9294	~5.1059
Overall survival time					
Age	0.022	0.4883	0.9846	0.9422	~1.0288
1772 C > T genotype	0.300	0.5908	0.8508	0.4722	~1.5330
T-stage	0.446	**0.0017**	4.0350	1.6850	~9.6624
N-stage	0.589	0.8594	1.1099	0.3502	~3.5181
Microvessel density	0.646	**0.0052**	6.0924	1.7175	~21.6115
VEGF expression	0.610	0.9791	0.9841	0.2979	~3.2517
HIF-1α expression	0.481	0.3225	1.6094	0.6269	~4.1315

^a^by Cox regression. SE, standard error; Exp. (B), exponent (B); CI, confidence interval.

^b^Bold numbers indicate significance.

## Discussion

The SNP 1772 C > T of HIF-1α gene chosen in current study are located within ODD of the HIF-1α. We found that T allele of the SNP 1772 C > T (P582S) of HIF-1α gene was significantly higher in 96 breast cancer patients than in 120 controls. In contrast, the association results of SNP 1772 C > T of HIF-1α gene with different kinds of cancers were not consistent in literature review. For example, the SNP 1772 C > T of HIF-1α gene was detected in several cancers [18-21,23] but it was absent for colorectal [34], and cervical [35] cancers.

Within ODD of the HIF-1α, proline residues 402 and 564 were reported to independently determine tightly binding to the von Hippel-Lindau (VHL) protein for HIF-1α ubiquitination and degradation under nonhypoxia condition [17,36-39]. In current study, however, the proline residue 582 located within ODD of the HIF-1α, i.e., the SNP 1772 C > T, was unable to interfere the binding of HIF-1α with VHL and to impair HIF-1α prolyl hydroxylation [40]. Similarly, the genotypes of SNP 1772 C > T of HIF-1α gene did not show significant difference between low and high HIF-1α levels in terms of immunostaining (Table 2). Other study [41] found that the HIF-1α overexpressed in immunostaining measurement for invasive breast cancer in the absence of 1772 C > T transition of HIF-1α gene. Accordingly, the role of SNP 1772 C > T of HIF-1α gene in its protein expression level is not clear. In future, the examination of more expression patterns of HIF-1α protein in these patients may clearly investigate this relationship.

Furthermore, the genotypes of SNP 1772 C > T of HIF-1α gene are not significantly associated with clinicopathologic characteristics and clinical outcome of breast cancer (Table 2) although SNP 1772 C > T of HIF-1α gene confers significant association with breast cancer (Table 1). Similar results were reported in prostate cancer study [21]. Therefore, the SNP 1772 C > T of HIF-1α gene is a good predictor for breast cancer risk but may be a poor clinicopathologic-associated factor.

The relationship between expression levels of HIF-1α and survival of breast cancer patients has been investigated. For example, high levels of HIF-1α were reportedly associated with decreased overall survival (*p* = 0.059) and disease-free survival (*p* = 0.110) [42]. Similarly, we found that HIF-1α expression shows the association with disease-free survival (*p* = 0.0732) but weak association with overall survival (*p* = 0.3225) (Table 3). These results suggest that expression levels of HIF-1α may be the potential risk factor for survival prediction of breast cancer.

The phenomena mentioned above may be partly explained by the multigene theory for carcinogenesis [43]. Furthermore, many SNPs may be associated with breast cancer. Although only single SNP was examined in our study, the SNP-SNP interaction [9,44-48] tumor may play a joint effect to associate with cancer and it is warranted for further investigation for multiple SNPs in breast cancer association.

## Conclusion

Taken together, SNP 1772 C > T (P582S) of HIF-1α gene confers significant association with breast cancer risk but it show no association with the clinicopathologic features and survival of breast cancer patients.
